# Albumin-to-alkaline phosphatase ratio may be a better predictor of survival than sclerostin, dickkopf-1, osteopontin, osteoprotegerin and osteocalcin

**DOI:** 10.1016/j.heliyon.2024.e29639

**Published:** 2024-04-12

**Authors:** K. Mathold, R. Nobin, L. Brudin, M. Carlsson, P. Wanby

**Affiliations:** a Department of Primary Care, Kalmar, Sweden; b Department of Orthopedics, Kalmar, Sweden; c Department of Clinical Physiology, Kalmar and Department of Medical and Health Sciences, University of Linköping, Sweden; d Department of Clinical Chemistry, Kalmar and Department of Medicine and Optometry, Linnaeus University, Sweden; e Department of Internal Medicine, Section of Endocrinology, Kalmar, Department of Medical and Health Sciences, University of Linköping and Department of Medicine and Optometry, Linnaeus University, Sweden

**Keywords:** Bone turnover marker, Albumin-to-ALP ratio, Sclerostin, Mortality, Cardiovascular disease, Fracture

## Abstract

**Objectives:**

The value of biochemical markers of bone turnover (BTMs) in predicting survival and disease remains unclear. In a prospective study we evaluated the novel biomarkers for bone turnover sclerostin, dickkopf-1 (DKK-1), osteopontin (OPN), osteoprotegerin (OPG) and osteocalcin (OC), as well as a traditional biomarker, alkaline phosphatase (ALP) in relation to risk of mortality, cardiovascular events and fractures.

**Participants:**

and Methods:Routine blood tests and serum BTMs, including ALP, were analyzed in patients with hip fracture n = 97, stroke n = 71 and healthy volunteers n = 83 (mean age 86, 83 and 77, respectively), followed for 7 years. Hazard Ratios (HR) were calculated for mortality, cardiovascular events and fractures in relation to these biomarkers. After adding the albumin-to-ALP ratio (AAPR) a post hoc analysis was performed.

**Results:**

120 participants died during the study. In the entire group of patients and volunteers (n = 251) higher AAPR (HR 0.28, 95 % CI 0.14–0.59, p < 0.001) was associated with decreased mortality. OPN and OPG were associated with mortality risk only in the univariate statistical analysis. HR for high AAPR in relation to new cardiovascular events was borderline significant (HR 0.29, 95 % CI 0.08–1.06, p = 0.061). None of the examined biomarkers were associated with new fractures, nor with an increased risk of a new cardiovascular event.

**Conclusions:**

AAPR may be a better predictor of mortality than the more novel BTMs, and higher AAPR could be associated with longer life expectancy. Further studies should determine the clinical usefulness of AAPR as a biomarker of mortality and cardiovascular disease.

## 1 Introduction

In this study we investigated whether serum levels of five established biochemical markers of bone turnover (BTMs) could be used to predict fractures, cardiovascular events and mortality in a cohort consisting of elderly patients with stroke or fractures, and volunteers. These BTMs (sclerostin, dickkopf-1, osteopontin, osteoprotegerin and osteocalcin) are quite well studied in the context of bone turnover. Sclerostin and dickkopf-1 (DKK-1) are released primarily by osteocytes, and inhibit bone formation by inhibiting WNT signalling [[1], [2], [3]]. Osteopontin (OPN) is an extracellular matrix protein released from several cell types to facilitate bone resorption [4]. Osteoprotegerin (OPG) is expressed by osteocytes and osteoblasts, and can reduce production of osteoclasts, and hence inhibit bone resorption [5]. Osteocalcin (OC) is a hydroxyapatite-binding protein of the bone matrix secreted by osteoblasts [6]. The levels of BTMs are known to change already after a few days or weeks following antiosteoporotic treatment [7,8]. BTMs may thus be useful to monitor the effect of treatment in osteoporosis, and may also serve as diagnostic tools.

In addition to the effect of bone turnover, experimental data also indicate that BTMs contribute to the development of atherosclerosis [9]. OPG appears more useful for the detection of early disease, while OPN is recruited in vessels after the establishment of disease. OC might constitute a marker to monitor response to interventional vascular treatments and risk of restenosis [10]. It is known that some of these BTMs increase with age [11,12], and in one study low DKK-1 was associated with mortality [13].

Whether alkaline phosphatase (ALP), a more traditional marker of bone turnover, might be useful as a biomarker of increased risk of fractures, cardiovascular events or mortality was examined as a secondary aim of the study. In a large systematic review and meta-analysis from 2019 Rahmani et al. found that high levels of liver parameters such as GGT, ALP and AST/ALT were associated with an increased CVD mortality rate [14]. Besides being expressed in the liver, isoforms of ALP are also expressed in bone tissue.

In recent years the albumin-to-alkaline phosphatase ratio (AAPR) has gained interest as a promising marker of prognosis in various cancers. According to a meta-analysis performed by An et al. decreased AAPR was associated with an adverse prognosis in cancer patients regarding overall survival, cancer-specific survival, disease-free survival and progression-free survival [15]. Decreased AAPR is a result of low levels of albumin and high levels of ALP. Low albumin reflects poor nutritional status, inflammation or liver- or kidney dysfunction [16,17]. To the best of our knowledge AAPR has not been studied in the context of medical conditions other than cancers.

The aim of the present study was to investigate if these five BTMs were associated with risk of future fractures, cardiovascular events or high mortality in a cohort of patients with fractures or stroke, and volunteers. The intention was to study the groups separately, but the numbers of patients in each group were quite low. We therefore chose to study the three groups together to increase statistical power. Deviant circulating levels of BTMs at baseline were hypothesized to be associated with increased mortality, increased risk of new cardiovascular events or new fractures. As a secondary aim we investigated if ALP could be used as a biomarker for these events, and in a post hoc analysis we also added AAPR.

## 2 Material and methods

### 2.1 Subjects

In this prospective study we followed the study population from a previous study on vitamin D. For details of study design see Carlsson et al. [18]. Briefly, between February 2014 and January 2015, 97 patients in their 75th year or older (≥74 years) admitted to the department of Orthopedics at the County Hospital of Kalmar, Sweden for sustained low-energy hip fracture, and another 71 patients ≥74 years with a new stroke diagnosed at the department of Medicine in the same hospital, were included in the study. 83 healthy volunteers ≥74 years without dementia, low-energy fracture, stroke or diabetic medication in their medical history were enrolled in the control group. These elderly volunteers considered themselves in good health despite their age. Weight and height were measured with the subjects barefoot in light clothing, and Body Mass Index (BMI) was calculated as kilograms per square meter. All participants gave their informed consent to participate in the study, which had received the approval of the Regional Ethical Committee, Linköping, Sweden.

After 7 years (SD 0.3) all participants were followed up concerning death, new low-energy fractures (at any skeletal location) or new cardiovascular events (fatal and non-fatal) by using the electronic medical record system Cambio Cosmic after additional ethical approval. Only a first new fracture or cerebrovascular event was recorded in the same patient. Cardiovascular events were defined as ICD-10 codes I21, I22, I20.0, I24.8, I24.9, I61, I64, G45 and G45.3. Patient records in the X-ray system SECTRA were searched to identify patients with new fractures.

### 2.2 Assays

Non-fasting venous blood samples were drawn early in the morning (7:00–9:00 a.m.) from all the included hip fracture patients. The blood samples from patients were obtained 3.8 (0–6) days upon hospitalization for hip fracture, and in most cases, 3 days following surgery. In the group with stroke patients, blood tests were drawn within 72h of the event. Blood samples from the volunteers were also obtained non-fasting. Analyses were performed at the Kalmar County, Clinical Chemistry Hospital Laboratory, and all steps were performed according to the manufacturers’ recommended protocol. Bone marker analyses were performed simultaneously using technology with multiples beads. For details regarding this assay please see Ref. [19]. Coefficients of variation (% CVs) for low and high concentrations, respectively, in the control material were <5 % for all BTMs.

Blood samples were analyzed for plasma calcium, albumin, Alkaline Phosphatase (ALP), and creatinine using the AU680, clinical chemistry analyzer from Beckman Coulter (Brea, CA USA). Estimated glomerular filtration rate (eGFR) was estimated from plasma creatinine using the revised Lund-Malmö GFR estimating equation.

Blood HbA1c were analyzed using Cobas c501 chemistry analyzer by Roche Diagnostics, Basel, Switzerland. Serum Ionized calcium was analyzed using ABL 825 (Radiometer Medical ApS, Bronshoj, Denmark).

### 2.3 Statistical analysis

STATISTICA (version 12, Statsoft, Tulsa, USA) was used for all statistical analyses. Descriptive statistics with mean values and standard deviation (SD) and median values and range were used for continuous variables. Categorical variables are presented as percentages. Osteocalcin was normally distributed, but the other four BTMs were not, and therefore their logarithms were used. Hazard Ratios (HR) were calculated with a corresponding confidence interval, in a Cox regression model (adjusted for differences in age, gender and patient group) for new fractures, new cardiovascular events, and mortality risk in participants with BTMs, ALP, AAPR and GFR. The continuous variables were transformed to their quartiles and these were then used as continuous variables in the model. This was done in order to minimize the number of statistical analysis and also to avoid outliers and to be able to judge if the trend was interupted at some quartile. Spearman correlation coefficients were determined for BTMs and ALP in relation to orienting blood samples and anthropometric measurements. P-values <0.05 were considered significant.

## 3 Results

The clinical characteristics of the study population and baseline levels of BTMs are summarized in Table 1. A total of 251 persons (94 men and 157 women) in the present study were followed up after 7 years (SD 0.3). 71 patients with stroke (33 men and 38 women; mean age 83.3 years) and 97 patients with a hip fracture were included (28 men and 69 women; mean age 85.8 years at baseline). 83 healthy volunteers (33 men and 50 women; mean age 77.3 years) were also included. The patient group members were older than the volunteers (p < 0.001). Patients with a stroke or fracture had higher serum levels of OPN, OPG and DKK-1 but lower levels of OC at baseline compared with healthy volunteers. Serum levels of sclerostin were significantly higher in the stroke group. The level of ALP was significantly higher in patients with fractures (p < 0.001) or stroke (p < 0.001) compared with volunteers. Albumin was lower in patients with stroke (p < 0.001) or fractures (p < 0.001) than in volunteers. AAPR was lower in patients with fractures (p < 0.001) or stroke (p < 0.001) compared with volunteers.

**Table 1:** Patient details. Healthy controls (C), orthopaedic patients with fragility fractures (F), and stroke patients (S). P-values from Mann-Whitneys *U* test provided Kruskal-Wallis nonparametric ANOVA was statistically significant (p < 0.05).

	Controls (C)	Fractures (F)	Stroke (S)	Total
**N**	83	97	71	251
**Gender**				
**Males (n; %)**	33 (40)	28 (29)	33 (46)	94 (37)
**Females (n; %)**	50 (60)	69 (71)	38 (54)	157 (63)
**Age (years)**				
**Mean (SD)**	77.3 (2.8)	85.8 (5.5)	83.3 (5.7)	82.3 (6.0)
**Median (Range)**	77 (74–89)	85 (76–97)	83 (75–97)	81 (74–97)
**p-value vs ctrls**	–	<0.001	<0.001	–
**BMI (kg/m**^**2**^**)**				
**Mean (SD)**	24.6 (3.2)	25.1 (4.7)	25.3 (4.6)	25.0 (4.2)
**Median (Range)**	24.5 (16.0–35.2)	24.6 (15.4–39.4)	25.4 (15.2–38.7)	24.7 (15.2–39.4)
**p-value vs ctrls**	–	–	–	–
**Systolic blood pressure (mmHg)**				
**Mean (SD)**	131.4 (14.0)	131.7 (15.9)	154.1 (21.2)	138.0 (19.8)
**Median (Range)**	130 (93–160)	130 (100–175)	155 (95–195)	140 (93–195)
**p-value vs ctrls**	–	0.834	<0.001	–
**Diastolic blood pressure (mmHg)**				
**Mean (SD)**	69.2 (8.3)	68.8 (8.9)	76.8 (13.2)	71.2 (10.7)
**Median (Range)**	70 (50–90)	70 (50–90)	80 (40–110)	70 (40–110)
**p-value vs ctrls**	–	0.505	<0.001	–
**Hb_A1c mmol/mol**				
**Mean (SD)**	37.5 (3.2)	39.6 (10.2)	43.9 (11.4)	40.1 (9.3)
**Median (Range)**	37 (30–51)	38 (28–119)	40 (31–103)	38 (28–119)
**p-value vs ctrls**	–	0.188	<0.001	–
**GFR ml/min/1.73 m**^**2**^				
**Mean (SD)**	59.9 (2.8)	51.7 (13.1)	54.2 (11.0)	55.1 (10.7)
**Median (Range)**	61 (46–61)	61 (10–61)	61 (16–61)	61 (10–61)
**p-value vs ctrls**	–	<0.001	<0.001	–
**ALP μkat/L**				
**Mean (SD)**	1.06 (0.27)	1.77 (0.88)	1.28 (0.45)	1.40 (0.69)
**Median (Range)**	1.00 (0.60–1.80)	1.60 (0.70–6.10)	1.20 (0.70–4.10)	1.20 (0.60–6.10)
**p-value vs ctrls**	–	<0.001	<0.001	–
**P–Ca (mmol/L)**				
**Mean (SD)**	2.29 (0.07)	2.15 (0.12)	2.31 (0.11)	2.24 (0.12)
**Median (Range)**	2.29 (2.10–2.48)	2.15 (1.90–2.68)	2.30 (2.11–2.73)	2.26 (1.90–2.73)
**p-value vs ctrls**	–	<0.001	0.203	–
**S-free_Ca (mmol/L)**				
**Mean (SD)**	1.25 (0.03)	1.22 (0.07)	1.26 (0.06)	1.24 (0.06)
**Median (Range)**	1.25 (1.18–1.36)	1.21 (1.06–1.55)	1.25 (1.16–1.48)	1.24 (1.06–1.55)
**p-value vs ctrls**	–	<0.001	0.761	–
**Albumin (mmol/L)**				
**Mean (SD)**	42.2 (2.1)	31.2 (3.4)	40.0 (3.9)	37.3 (5.9)
**Median (Range)**	42.0 (37.0–47.0)	31.0 (24.0–39.0)	40.0 (32.0–49.0)	39.0 (24.0–49.0)
**p-value vs ctrls**	–	<0.001	<0.001	–
**Albumin/ALP-ratio**				
**Mean (SD)**	42.5 (11.3)	21.0 (8.9)	34.0 (9.7)	31.8 (13.5)
**Median (Range)**	41.0 (22.8–70.0)	19.7 (5.1–48.8)	33.3 (9.3–60.0)	30.8 (5.1–70.0)
**p-value vs ctrls**	–	<0.001	<0.001	–
**Osteopontin μg/L (OPN)**				
**Mean (SD)**	20368 (6937)	81471 (52582)	37447 (20345)	48813 (43732)
**Median (Range)**	20078 (3610–40496)	68549 (13613–387553)	36329 (5430–108799)	36329 (3610–387553)
**p-value vs ctrls**	–	<0.001	<0.001	–
**Osteocalcin μg/L (OC)**				
**Mean (SD)**	9452 (3646)	5916 (3554)	7674 (4353)	7588 (4094)
**Median (Range)**	9482 (2260–21897)	5135 (195–15992)	6625 (1986–26772)	7113 (195–26772)
**p-value vs ctrls**	–	<0.001	<0.001	–
**Osteoprotegerin ng/L (OPG)**				
**Mean (SD)**	205.2 (65.7)	522.1 (195.1)	481.0 (273.2)	406.5 (239.2)
**Median (Range)**	205 (61–354)	504 (216–1346)	435 (110–2302)	372 (61–2302)
**p-value vs ctrls**	–	<0.001	<0.001	–
**Sclerostin μg/L**				
**Mean (SD)**	2466 (1216)	2545 (1405)	3315 (1594)	2738 (1446)
**Median (Range)**	2280 (207–6457)	2357 (319–7354)	3138 (571–8746)	2460 (207–8746)
**p-value vs ctrls**	–	0.829	<0.001	–
**Dickkopf-1 ng/L (DKK-1)**				
**Mean (SD)**	189.6 (94.6)	302.5 (157.1)	287.5 (159.6)	260.9 (148.7)
**Median (Range)**	172 (63–521)	267 (84–733)	253 (64–953)	213 (63–953)
**p-value vs ctrls**	–	<0.001	<0.001	–

Sixty-eight percent of the patients and 6 % of the volunteers died during the follow-up period. Patient group, age, BMI and HbA1c were all associated with higher mortality (Table 2). According to the multivariate statistical analysis the risk of dying was approximately 4 times higher in the fracture group compared with volunteers (HR 4.3, 95 % CI 1.5–12.7, p = 0,007), and more than 6 times higher in the stroke group compared with volunteers (HR 6.8, 95 % CI 2.6–18.0, p < 0,001). BMI >35 and high HbA1c were correlated with increased mortality. In the univariate Cox regression analysis we found an association between OPN, OPG, ALP, AAPR (but not albumin alone), GFR and mortality, but after multivariate statistical analysis AAPR and GFR remained as the only predictors of survival. HR for mortality was 0.28 (95 % CI 0.14–0.59, p < 0,001) in the highest quartile of AAPR compared with the lowest. HR for mortality was 0.52 (95 % CI 0.30–0.89, p 0.018) in individuals with GFR >60 ml/min compared with those with GFR ≤ 30 ml/min. For further details see Table 2.

**Table 2:** Hazard rate ratios (HR) for all deaths analyzed using Cox regression. Univariate (adjusting for age, gender and patient group) to the left and multivariate to the right.

Parameter		All deaths		Univariate Cox regression		Multivariate Cox regression	
	Total	n	(%)	HR (95 % CI)	p	HR (95 % CI)	p
Patient group							
**Control**	83	5	6	1.00		1.00	
**Fracture**	97	70	72	8.7 (3.3–22.9)	<0.001	4.3 (1.5–12.7)	0.007
**Stroke**	71	45	63	8.7 (3.4–22.6)	<0.001	6.8 (2.6–18.0)	<0.001
**Age category (yrs)**							
**<77**	72	10	14	1.00		1.00	
**77**–**82**	70	27	39	1.41 (1.14–1.75)		1.35 (1.09–1.68)	
**83**–**87**	54	35	65	1.99 (1.30–3.05)		1.83 (1.18–2.83)	
**>87**	55	48	87	2.81 (1.49–5.32)	0.001	2.47 (1.28–4.77)	0.007
**Gender**							
**Female**	157	79	50	1.00		1.00	
**Male**	94	41	44	1.23 (0.83–1.83)	0.306	1.33 (0.88–1.99)	0.172
**BMI (kg/m**^**2**^**)**							
**<25**	131	66	50	1.00		1.00	
**25**–**34.9**	115	52	45	0.61 (0.43–0.87)		0.60 (0.42–0.86)	
**>=35**	5	2	40	0.37 (0.18–0.75)	0.006	0.36 (0.18–0.74)	0.006
**Blood pressure**							
**SBP<=120**	64	33	52	1.00		–	
**Normal**	94	38	40	0.8 (0.7–1.1)		–	–
**SBP>140_or_DBP>90**	77	37	48	0.71 (0.43–1.15)		–	–
**SBP>=170**	16	12	75	0.59 (0.29–1.23)	0.160	–	–
**HbA1c (mmol/mol)**							
**<=34**	37	17	46	1.00		–	–
**34**–**41**	147	53	36	1.15 (0.93–1.42)		–	–
**42**–**48**	43	32	74	1.32 (0.87–2.02)		–	–
**>48**	21	15	71	1.52 (0.81–2.88)	0.194	–	–
**GFR**							
**<=30**	13	9	69	1.00		1.00	
**30**–**60**	76	51	67	0.74 (0.56–0.97)		0.72 (0.55–0.95)	
**>60**	162	60	37	0.55 (0.32–0.95)	0.031	0.52 (0.30–0.89)	0.018
**ALP**							
≤1	50	11	22	1.00		–	
1.1-1.2	81	35	43	1.25 (1.02–1.55)		–	–
1.3-1.6	63	35	56	1.57 (1.03–2.39)		–	–
>1.6	54	38	70	1.97 (1.05–3.69)	0.034	–	–
**P_Ca_Q4**							
**<=2.16**	63	40	63	1.00		–	
**2.16-2.26**	72	34	47	1.15 (0.93–1.42)		–	–
**2.26-2.32**	59	20	34	1.32 (0.86–2.02)		–	–
**>2.32**	57	26	46	1.51 (0.80–2.88)	0.206	–	–
**S_Free_Ca_Q4**							
**<=1.21**	76	42	55	1.00		–	
**1.21-1.24**	66	29	44	1.10 (0.93–1.31)		–	–
**1.24.1.27**	50	22	44	1.22 (0.86–1.72)		–	–
**>1.27**	59	27	46	1.35 (0.80–2.26)	0.260	–	–
**P_alb_Q4**							
**<=32**	66	52	79	1.00			
**32**–**39**	70	42	60	0.80 (0.59–1.09)		–	
**39**–**42**	66	12	18	0.64 (0.35–1.18)		–	–
**>42**	49	14	29	0.51 (0.21–1.28)	0,152	–	–
**alb_ALP_Q4**						–	–
**<=22.3**	61	48	79	1.00		1.00	
**22.4**–**30.8**	63	35	56	0.66 (0.52–0.85)		0.66 (0.51–0.84)	
**30.8**–**40.9**	61	24	39	0.44 (0.27–0.72)		0.43 (0.26–0.70)	
**>40.9**	63	12	19	0.29 (0.14–0.60)	<0.001	0.28 (0.14–0.59)	<0.001
**Osteopontin_Q4**							
**<21851**	63	9	14	1.00		–	
**21852**–**36329**	63	22	35	1.31 (1.01–1.69)		–	–
**36329**–**62269**	63	45	71	1.70 (1.02–2.85)		–	–
**>62269**	62	44	71	2.22 (1.03–4.82)	0.042	–	–
**Osteocalcin_Q4**							
**<4646**	63	42	67	1.00		–	
**4646**–**7167**	62	29	47	0.98 (0.83–1.17)		–	–
**7178**–**9787**	61	24	39	0.97 (0.68–1.37)		–	–
**>9787**	62	23	37	0.95 (0.56–1.61)	0.856	–	–
**Osteoprotegerin_Q4**							
**<230**	63	8	13	1.00		–	
**231**–**367**	62	20	32	1.37 (1.07–1.75)		–	–
**368**–**535**	63	44	70	1.87 (1.14–3.05)		–	–
**>535**	62	48	77	2.55 (1.22–5.32)	0.013	–	–
**Sclerostin_Q4**							
**<=1656**	63	34	54	1.00		–	
**1657**–**2459**	62	19	31	0.98 (0.80–1.19)		–	–
**2460**–**3703**	63	31	49	0.96 (0.64–1.43)		–	–
**>3703**	62	36	58	0.93 (0.51–1.70)	0.824	–	–
**DKK_Q4**							
**<=164**	63	20	32	1.00		–	
**165**–**213**	63	27	43	1.16 (0.98–1.38)		–	–
**214**–**316**	63	31	49	1.35 (0.96–1.90)		–	–
**>316**	62	42	68	1.56 (0.94–2.61)	0.086	–	–

Five patients were lost in the follow-up. In total we registered 32 new cardiovascular events, 9 myocardial infarctions and 26 strokes, during the follow-up period. None of the BTMs were associated with increased risk of a new cardiovascular event. The risk of having a new cardiovascular event was higher in patients with impaired renal function. HR for a new cardiovascular event was 0.31 (95 % CI 0.10–0.97, p = 0.044) in research participants with GFR >60 ml/min compared with those with GFR ≤ 30 ml/min. The HR for a new cardiovascular event was 0.29 (0.08–1.06, p = 0.061) in individuals with AAPR in the highest versus the lowest quartile. The risk of suffering a new cardiovascular event was significantly higher in the stroke group compared with the other groups.

There was a total of 49 new fractures registered in the three groups altogether. None of the BTMs, ALP, GFR or AAPR correlated with a new fracture. The only parameters associated with increased risk of a new fracture were patient group and gender. The risk of new fractures increased threefold in the fracture group (HR 3.2, 95 % CI 1.2–8.2, p = 0.015) compared with the volunteers, and women had a reduced risk of suffering a new fracture compared to men (HR 0.32, 95 % CI 0.15–0.71, p = 0.005).

A positive correlation was seen between ALP and DKK-1 (r = 0.238, p < 0.001), OPG (r = 0.443 p < 0.001) and OPN (r = 0.502, p < 0.001). There was a significant correlation between OPN, Sclerostin, DKK-1, OPG and GFR. For further details concerning correlations see Table 3.

**Table 3:** Correlations using Spearman rank order correlation. r is the correlation coefficient and p the statistically significance level.

Variable	Sex	Age	BMI	HbA1c	GFR	ALP	Ca	Free_Ca	Albumin	OPN	OC	OPG	Sclerostin	DKK
Sex (female = 1; male = 2)														
**r**	–	−0.15	0.10	0.07	−0.07	−0.07	0.06	0.15	0.21	−0.05	0.05	−0.01	0.48	0.05
**p**	–	0.021	0.097	0.270	0.239	0.252	0.328	0.016	0.001	0.407	0.396	0.856	<0.001	0.409
**Age (years)**														
**r**		–	−0.10	0.11	−0.23	0.31	−0.27	−0.15	−0.54	0.53	−0.25	0.61	0.01	0.28
**p**		–	0.111	0.094	<0.001	<0.001	<0.001	0.018	<0.001	<0.001	<0.001	<0.001	0.855	<0.001
**Body mass index (BMI; kg/M**^**2**^**)**														
**r**			–	0.18	−0.11	−0.02	0.05	0.01	0.07	0.00	−0.18	−0.04	0.15	0.12
**p**			–	0.004	0.095	0.756	0.389	0.854	0.244	0.965	0.005	0.543	0.015	0.063
**Glycohemoglobin, hemoglobin A1c (HbA1c; mmol/mol)**														
**r**				–	−0.23	0.04	0.03	0.01	−0.02	0.10	−0.09	0.21	0.24	0.17
**p**				–	<0.001	0.537	0.652	0.891	0.794	0.123	0.162	0.001	<0.001	0.006
**Glomerulis filtration rate (GFR ml/min/1.73 m2)**														
**r**					–	−0.17	0.20	0.07	0.25	−0.35	−0.03	−0.42	−0.31	−0.36
**p**					–	0.009	0.002	0.296	<0.001	<0.001	0.675	<0.001	<0.001	<0.001
**Alkaline phosphatase (ALP μkat/L)**														
**r**						–	−0.25	−0.17	−0.44	0.50	−0.15	0.44	−0.01	0.24
**p**						–	<0.001	0.008	<0.001	<0.001	0.019	<0.001	0.886	<0.001
**P-calcium (mmol/L)**														
**r**							–	0.75	0.68	−0.45	0.24	−0.36	0.08	−0.15
**p**							–	<0.001	<0.001	<0.001	0.063	<0.001	0.228	0.020
**S-free Calcium (mmol/L)**														
**r**								–	0.42	−0.28	0.24	−0.21	0.16	−0.08
**p**								–	<0.001	<0.001	<0.001	0.001	0.010	0.188
**P-albumin (mmol/L)**														
**r**									–	−0.69	0.35	−0.57	0.11	−0.25
**p**									–	<0.001	<0.001	<0.001	0.096	<0.001
**Osteopontin μg/L (OPN)**														
**r**										–	−0.18	0.67	0.06	0.35
**p**										–	0.004	<0.001	0.355	<0.001
**Osteocalcin μg/L (OC)**														
**r**											–	−0.27	−0.03	−0.24
**p**											–	<0.001	0.637	<0.001
**Osteoprotegerin ng/L (OPG)**														
**r**												–	0.32	0.53
**p**												–	<0.001	<0.001
**Sclerostin μg/L**														
**r**													–	0.36
**p**													–	<0.001
**Dickkopf-1 ng/L (DKK-1) (DKK)**														
**r**														–
**p**														–

## 4 Discussion

Biomarkers to predict prognosis in common, serious diseases such as osteoporosis and cardiovascular disease are important in order to properly examine and correct risk factors in individuals identified with poor prognosis. In this prospective study following a cohort consisting of one group of hip fracture patients, one group of stroke patients and one group of volunteers we found, after 7 years, that low GFR, high ALP, low AAPR, high OPN and high OPG at baseline were associated with an increased mortality risk. However, after a multivariate statistical analysis only low AAPR and low GFR remained significantly associated with increased mortality. We found no association between any of the examined biomarkers and increased risk of new fractures. Low GFR was as expected also associated with increased risk of new cardiovascular events, and there was a trend towards high AAPR being associated with a lower risk of new cardiovascular events.

AAPR and GFR were the best prognostic markers of mortality in our study. Impaired renal function is a serious medical condition with a well-established association with increased mortality and cardiovascular disease [20]. Our findings regarding AAPR and mortality are in line with the results of some studies of various cancers [15]. However, AAPR has not been studied in other medical conditions to the best of our knowledge. Decreased AAPR is explained by low albumin and high ALP. Albumin is an acute phase reactant and decreases as a response to tissue damage and inflammation. Other possible causes of low albumin are liver disease or kidney disease [16,17]. High ALP was associated with increased mortality in our study in a univariate statistical analysis. The finding of an association between ALP and mortality is in line with the results from the meta-analysis performed by Rahmani et al. [14]. In their study the increased mortality was reported to be due to cardiovascular disease. There are several other studies demonstrating an association between ALP and cardiovascular disease and/or mortality [[21], [22], [23], [24]]. ALP might increase vascular calcification and possibly promote inflammation, since an association between C-reactive protein (CRP) and ALP has been observed [25]. However, there was no association between levels of ALP and risk of new cardiovascular events in our study, but a borderline significant association between AAPR and new cardiovascular events.

Both sclerostin and DKK-1 are inhibitors of the Wnt-signalling pathway, and thereby also of bone formation [26]. Wnt signalling is not limited to bone tissue, and DKK-1 expression has been demonstrated in murine atherosclerotic lesions and human carotid plaques [9]. One study found clearly increased serum levels of sclerostin and DKK-1 (also of OPG and OPN) in patients with stroke compared with volunteers [27]. Dovjac and colleagues demonstrated low serum levels of DKK-1 associated with mortality [28], and in a newly published study by Klingenschmid and colleagues they showed that elevated baseline serum levels of DKK-1 were associated with incident cardiovascular events [29]. Interestingly romosozumab, which is a recently approved therapy against osteoporosis acting by inhibiting sclerostin, exhibit cardiovascular events as side effects [30]. In clinical praxis a patient history of myocardial infarction or stroke therefore constitutes contraindications for the use of romosozumab. However, we found no association between sclerostin and increased risk of cardiovascular events, deaths or fractures: and the same was true for DKK-1.

OPN is a non-collagenous protein involved in bone matrix organization and deposition. It is a marker of both bone formation and bone resorption. OPN is a multifunctional protein that is expressed by several cell types including osteoblasts and osteocytes. In a study regarding patients with coronary artery disease high OPN levels were a strong predictor of all-cause death [31]. In our study we found a correlation between OPN and increased mortality in the univariate statistical analysis, but not with new cardiovascular events or fractures.

OPG inhibit osteoclast formation and activity [32]. There are several studies supporting an association between elevated circulating levels of OPG and increased risk of cardiovascular events [33,34], and also with geriatric frailty syndrome [35]. It has been hypothesized that circulating OPG levels rise due to transition of vascular smooth muscle cells to osteoblast-like cells in atherosclerosis and might reflect mechanisms to counteract vascular calcification [36,37]. Interestingly, we found no association between OPG and cardiovascular events, but a significant association with mortality in the univariate statistical analysis. The use of OPG in fracture risk prediction and monitoring osteoporosis treatment has been investigated, but results have been inconsistent [32]. We found no association between fracture risk and OPG.

OC is a small non-collagenous protein synthesized by osteoblasts and odontoblasts. After carboxylation the altered protein can bind with hydroxyapatite and mineralize the bone matrix [32]. OC is excreted by the kidneys and is one of the most abundant non-collagenous proteins in bone. OC has been shown to have a negative correlation with hip fracture in elderly women [1]. In the present study we could not find any association between OC levels and increased risk of fractures, cardiovascular events or mortality.

A strength of the present work is the simultaneous study of several novel BTMs, and also in relationship to ALP, GFR and AAPR. AAPR is calculated based on easily accessible analyses in most labs, which makes AAPR a promising candidate in the search for suitable biomarkers. Another advantage of the present study is that the follow-up interval was quite long (7 years).

We acknowledge several limitations of our study. A weakness is that the blood samples were drawn non-fasting, and previous studies have shown that food intake results in a decrease in some BTMs. The circadian rhythm is also important, and in the patient groups all blood tests were drawn in the morning, but in the group of volunteers during the day. This is particularly relevant for bone resorption markers that show higher levels in the second half of the night and upon waking [26].

Another disadvantage of our study is that we were unable to determine the origin of ALP. ALP exists in several isoforms, which emanates from the liver or skeleton. We observed a correlation between ALP and some parameters related to bone tissue such as certain BTMs (DKK-1, OPG, OPN), and also a weak correlation between serum-free calcium and ALP. Unfortunately we did not analyze liver parameters.

We chose to add AAPR as a variable post hoc, which is also a limitation of the study. Further studies with AAPR as a primary scientific question are needed.

Another weakness of our study is the fact that the number of new cardiovascular events were quite few, which might have affected our results. Cardiovascular events are the leading cause of death, but autopsies are seldom performed in Sweden, and therefor some cardiovascular events probably went undetected. We were also quite strict in our definition of new cardiovascular events and only registered acute cardiovascular events requiring hospitalization.

## 5 Conclusion

In the present study we investigated whether the BTMs sclerostin, OPG, OPN, OC and DKK-1 could be useful as biomarkers to predict increased risk of new fractures, new cardiovascular events or mortality in a cohort of elderly patients with fractures, stroke and volunteers. We compared the BTMs with more traditional biomarkers such as ALP and GFR, and added AAPR in a post hoc analysis. In summary, both a decreased AAPR and a decreased GFR were found to be better predictors of mortality than the examined BTMs. We found no association between any of the studied markers and risk of future fractures. AAPR, previously not studied in the context of medical conditions other than cancers, may be useful in predicting new cardiovascular events, but its predictive value versus GFR needs to be established. However, to determine a possible role of AAPR as a risk marker of cardiovascular events and of mortality we call for larger studies in diverse patient populations.

## Funding

This work was supported by a Grant from the Kamprad family foundation for entrepreneurship, research and charity.

## Data availability statement

Data can on request to the corresponding author be made available.

## Ethics declarations

This study was reviewed and approved by the Regional Ethical Committee of Linköping, Sweden, with the approval numbers: Dnr 2013/404-31 + Dnr 2016/351-31 and by the Regional Ethical Committee of Uppsala, Sweden, with the approval number 2019–05054.

All participants/patients (or their proxies/legal guardians) provided informed consent to participate in the study.

## CRediT authorship contribution statement

**K. Mathold:** Conceptualization, Investigation, Writing – original draft, Writing – review & editing. **R. Nobin:** Conceptualization, Investigation, Writing – original draft. **L. Brudin:** Conceptualization, Methodology, Formal analysis. **M. Carlsson:** Conceptualization, Investigation, Methodology. **P. Wanby:** Conceptualization, Formal analysis, Funding acquisition, Methodology, Project administration, Resources, Writing – original draft, Writing – review & editing, Investigation.

## Declaration of competing interest

The authors declare no conflict of interest.
